# Locomotor Activity Monitoring in Mice to Study the Phase Shift of Circadian Rhythms Using ClockLab (Actimetrics)

**DOI:** 10.21769/BioProtoc.5187

**Published:** 2025-02-20

**Authors:** Andrea Brenna, Jürgen A. Ripperger, Urs Albrecht

**Affiliations:** 1 Section of Medicine, University of Fribourg, Fribourg, Switzerland; 2 Department of Biology, University of Fribourg, Fribourg, Switzerland

**Keywords:** Phase shift, Aschoff-type II, Aschoff-type I, Phase delay, Phase advance, Circadian rhythms, Nocturnal light

## Abstract

The circadian clock regulates biochemical and physiological processes to anticipate changes in light, temperature, and food availability over 24 h. Natural or artificial changes in white/blue lighting exposure (e.g., seasonal changes, jet lag, or shift work) can advance or delay the clock phase to synchronize physiology with the new environmental conditions. These changes can be monitored through behavioral experiments in circadian research based on the analysis of locomotor activity by measuring wheel-running revolutions. The protocol includes measuring the internal period length in constant darkness and administering nocturnal light pulses to mice kept either in light/dark conditions (LD 12:12, Aschoff-type II protocol) or continuous darkness (DD, Aschoff-type I). Here, we describe a step-by-step guide for researchers to analyze the mouse circadian clock using wheel-running experiments and ClockLab (Actimetrics) to quantify data.

Key features

• This protocol builds upon the method developed by Jud et al. [1], optimized for digital analysis using the ClockLab software.

• Step-by-step tutorial on measuring period length, analyzing periodograms, assessing general activity, and determining phase shifts (Aschoff Type I and II).

## Background

Biological rhythms are adaptive physiological and metabolic processes, allowing virtually all organisms to anticipate changes in a light/dark cycle occurring over 24 h [2]. These processes are oscillating physiological responses to exogenous stimuli, namely zeitgebers or time-givers [3], such as light, temperature, and nutrients. These oscillations, driven by the above-mentioned stimuli, are defined as diurnal rhythms. The perception of white/blue light is one of the most important mechanisms for entraining the circadian clock to engage in diurnal behaviors [4]. Diurnal rhythms are virtually observable in almost all living organisms. In the absence of external stimuli (i.e., light), living organisms display a self-sustained biological rhythm called circadian (Latin: Circa Diem, around a day) with an internal period (tau, τ) slightly shorter than 24 h [5,6]. Since the divergence between circadian and diurnal rhythms can affect the survival rate, as observed in mice [7], living organisms must be entrained in the light/dark cycle (L:D) daily. Rodents are a well-known model for studying circadian rhythms by analyzing their rhythmic wheel-running activity [8]. Mice kept in diurnal conditions spend more time running on the wheel at night. Mice kept in constant darkness, by shielding them from external illuminating cues, display so-called free running or circadian rhythms, which are self-sustained in the absence of additional stimuli [1]. Nocturnal light can reset the endogenous circadian rhythms through a process called phase shift (ф) [9]. The direction of the phase shift depends on the clock’s temporal state. Light perceived in the early night promotes phase delays (namely, a delay in the activity onset). On the other hand, a light pulse late at night promotes phase advances (namely, anticipation in the activity onset), whereas light in the middle of the day does not alter the clock phase. The phase shift is manifested at the behavioral level with a change of locomotor activity onset (phase shift) the day after the light pulse. Here, we show a detailed protocol used for analyzing and quantifying the wheel-running activity of mice using ClockLab (Actimetrics) to determine period length, general activity profile, and phase shift of the circadian clock. We became aware that a step-by-step protocol that can guide researchers to perform such articulate experiments in a user-friendly way is not available online. Therefore, we propose a detailed version of the previously published one [1].

## Materials and reagents

**Biological materials**

1. 3–6-month-old 129/C57BL6 mice

## Equipment

1. Wheel-running facility

a. Soundproof ventilated chambers at constant temperature (22 ± 2 °C) and humidity (40%–50%)

2. Wheel-running cages

a. Cage (280 mm long × 105 mm wide × 125 mm high) (Tecniplast, catalog number: 1155M)

b. Stainless steel wire lid (Tecniplast, catalog number: 1264C116)

c. Stainless running wheel (diameter 115 mm) (Trixie GmbH, catalog number: 6083)

d. Magnet (Fehrenkemper Magnetsysteme, catalog number: 34.601300702)

e. Magnetic switch: Reed-Relais 60 (Conrad Electronic AG, catalog number: 503835-22)

3. Mouse housing

a. Water bottles (260 mL, 55 × 128 mm, polycarbonate, with silicone ring) (Tecniplast, catalog number: ACBTO262)

b. Bottle caps (Tecniplast, catalog number: ACCP2521)

c. Nestlets (5 × 5 cm) (EBECO)

d. Animal bedding (Schill AG, model: Bedding type 3–4)

e. Chow food (Kliba-Nafag, catalog number: 3432PX)

4. Illuminating system

a. Light bulb, 18 W (Mazdafluor, model: Symphony AZURA 965)

b. Light bulb mounting (230 V, 50 Hz, 0.37 A) (Mazda, model: Mx204-118)

c. Fan: accessories (CF-1212, 12 V=/500 mA) (Monacor, catalog number: 03.1670)

d. Luxmeter (Testo, GmbH & Co, 0–100.000 lux)

5. Computer hardware and software

a. Microsoft Windows PC (e.g., Dell, Intel Pentium III running Windows 2000 or higher)

b. Data acquisition board: National Instruments AMUX 64-T (fitted with 10-kΩ resistors)

c. RJ45 socket

d. PCI 6503 card National Instruments

e. National Instruments NI-DAQ software

f. ClockLab software package, Actimetrics

*Note: ClockLab components can be purchased in a ready-to-use package from Actimetrics.*

## Procedure

**Figure 1.**
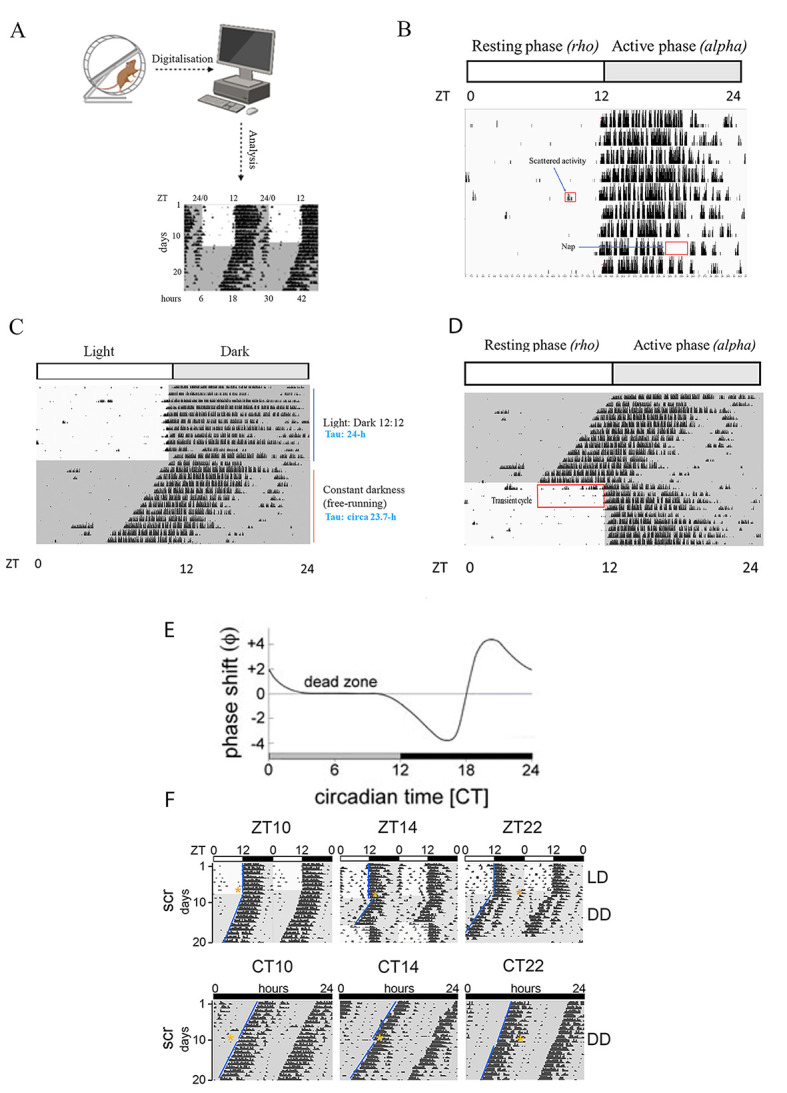
Digitalization and interpretation of actograms. **A)** Graphic representation of an actogram produced by ClockLab after elaborating on data obtained from the wheel-running cage and processed by the computer. The actogram is obtained from a published paper [16]. **B)** Representative single-plotted actogram displaying a mouse’s wheel-running activity showing activity bins (black vertical bars) concentrated at nighttime when mice are kept in 12:12 h light/dark conditions. The height of each vertical bar indicates the accumulated number of wheel revolutions for a given interval of time previously selected (e.g., 10 min). Each horizontal line corresponds to one day. The *rho*- and *alpha*-phases indicated on the top of the actogram refer to rest and activity, respectively. The red box in the light phase indicates the scattered activity of a mouse during the resting phase in the daytime. The red box in the dark phase indicates that the mouse is resting at nighttime. **C)** Representative single-plotted actogram shows the different distribution of mouse activity when it is kept in diurnal (12:12 h light/dark) or circadian (free running) conditions. The Tau (τ) in a 12:12 h light/dark condition is approximately 24 h, while the one displayed by mice kept in constant darkness is slightly shorter. Since the computer timer is set on the 24-h day cycle, the wheel-running activity in constant darkness looks like a slope. **D)** The single-plotted actogram displays in the red box the mouse activity in the daytime as a consequence of the re-entrainment from the constant darkness to the 12:12 h light/dark cycle. This event produces a transient cycle. **E)** The phase response curve (PRC) for nocturnal rodents, produced in circadian conditions (constant darkness) [1]. The gray and black bars below the PRC indicate subjective day and night, respectively. The X-axis shows the circadian time (CT) at which the researcher applied the light. The Y-axis displays the amplitude in hours of the phase shift (φ). Light pulses administered between CT11 and CT16 provoke a phase delay (negative values), whereas light pulses between CT19 and CT3 generate phase advances (positive values). A light pulse given between CT4 and CT10 does not produce any phase shift (dead zone). We can observe the inversion point in the phase shift response starting at CT16 and reaching the singularity (blue star) at CT18. **F)** The double-plotted actogram shows examples of a phase delay (middle actogram), phase advance (right actogram), and no delay (left actograms) following the Aschoff-type II (upper actograms, diurnal conditions) and Aschoff-type I protocol (lower actogram, circadian conditions). The yellow stars indicate when the light was applied. For more details, please check the original paper [16].

Below, we describe the step-by-step procedure for characterizing the internal period length of mice and measuring the phase shift of their circadian clock by employing the wheel-running method. This method is largely used for studying modifications in the endogenous circadian clock of mice, reflected in an altered free-running behavior. In this section, we will describe two different approaches, named Aschoff-type II (phase shift measured in animals kept in diurnal conditions) and Aschoff-type I (phase shift measured in animals kept in constant darkness) [10]. We provide a step-by-step protocol that will facilitate users to analyze the period length (τ) and phase shift (ф) of the circadian clock. However, before starting to describe the procedure, the reader should become familiar with the following concepts that will be subsequently discussed:

• **Actogram.** Circadian rhythmicity in rodents can be measured by analyzing a general output like locomotor activity. In this specific approach, a sensor is connected to a wheel-running cage, which transmits the signal to the computer. The ClockLab (Actimetrics) software subsequently elaborates the data, which are displayed on the computer monitor (Figure 1A). Vertical bars appearing on the computer monitor are the readout of the number of wheel revolutions per time, and this specific graph is called an actogram. Each horizontal line represents one day. The height of each vertical black bar cluster indicates the sum of wheel revolutions happening in a defined amount of time (i.e., 10’). Mice are nocturnal animals, therefore, when they are kept under 12:12 h light/dark cycles, their activity is concentrated at nighttime (*alpha*), while the resting activity is in the daytime (*rho*) (Figure 1B). Under the 12:12 h light/dark cycle, resembling the solar day, *alpha*- and *rho*-phases are opposite in diurnal and nocturnal organisms [11]. Sometimes, scattered activity can be observed in the *rho* phase because the animal interrupts its sleep for a short time (Figure 1B, red box in the *rho* phase).

• **Diurnal rhythm vs. free running.** Diurnal rhythms are tied to the external stimuli (Zeitgeber). When the external stimulus is a light pulse (white or blue), we can refer to the time taken into consideration for analyzing the locomotor activity as Zeitgeber time (ZT). Within the 12:12 h light/dark cycle, ZT0 is defined as *lights on*, the beginning of the light phase, and ZT12 corresponds to *lights off*, the end of the light phase. Diurnal rhythms are equal to 24 h, and as mentioned above, the wheel-running activity is concentrated at nighttime. On the other hand, organisms kept in constant darkness can display the so-called free-running period or circadian rhythms. These rhythms may persist indefinitely in the absence of any synchronizing cue. The period length of these free-running rhythms is often not equal to 24 and differs from species to species. For instance, wild-type mice (C57BL/6Tyrc-Brd × 129S7) have an internal period length of 23.7 ± 0.1 h [12]. While in diurnal rhythms the temporal unit is the hour (ZT), in the free running we talk about the circadian hour (CT), which consists of quantifying the internal period length (tau) and dividing it by 24. During the first day of constant darkness, we can assume ZT = CT. Since circadian rhythms are manifested in constant darkness, we can talk about subjective day (CT0-12) and subjective night (CT12-24), where physiological processes reflect the diurnal profile. Figure 1C shows an example of the difference between diurnal and circadian rhythms.

• **Entrainment.** This is a process promoted by specific stimuli, such as light, food, and temperature, that induces the alignment between the endogenous clock and the external environment [13]. For instance, mice kept in constant darkness exhibit an endogenous period shorter than 24 h. However, when these rhythms are adjusted to a 12:12 h light/dark cycle, the endogenous clock aligns with the 24-h environmental rhythm after a few days. This process promotes the masking effect of diurnal rhythms over circadian ones. When constant blue/white light is applied to entrain the animals, we talk about the *continuous mode*. On the other hand, a short-term stimulus (minutes) given during the night can promote a phase shift of the circadian clock (see below). In this specific case, we talk about the *phasic mode*. The environmental signals that can entrain circadian clocks are called Zeitgebers [3] (Figure 1D, constant darkness vs. re-entrained light/dark cycle). When mice kept in constant darkness are re-entrained to a diurnal rhythm, we can observe a transient cycle [14]. It can be observed as a wheel-running revolution during the *rho* phase anticipating the activity onset, which can persist for a few days (Figure 1D, red box). Those transient cycles reflect the disequilibrium between the altered *phase angle* generated by the endogenous circadian rhythm of mice, which is < 24 h, and the entrained rhythm to the Zeitgeber (24 h) [11]. With the term “phase angle” (Ψ) we refer to the relationship between the timing of the biological clock and the timing of an external time cue [14].

• **Phase shift.** Phase shift (ф) refers to a shift in the animal’s activity onset as a consequence of a white/blue light pulse given during the night [15]. The phase response curve (PRC), whose shape varies depending on species and stimulus [14], displays the phase shift profile over 24 h when a light pulse is given every hour with mice kept in constant darkness. Figure 1E shows a typical light PRC of a nocturnal rodent. The phase curve can be divided into three parts: a phase delaying zone of the activity onset (CT12 to CT16), a phase advancing zone (CT18 to CT2), and a dead zone (CT2 to CT8, subjective day), observed when the stimulus does not affect the activity onset phase. Between CT16 and CT18 is the so-called *singularity point* where the PRC crosses the baseline (Figure 1E, blue star). If light hits exactly at that point (which is difficult to achieve), arrhythmicity is induced in the animal. The phase shift of the circadian clock can also be observed in diurnal conditions and the effect resembles what is observed in constant darkness (Figure 1E and F).

*Note: Actograms are usually double plotted to show diurnal/circadian activity over multiple days, facilitating the identification of persisting rhythms (Figure 1F).*

**A. Mice preparation and experimental setup**

**Figure 2.**
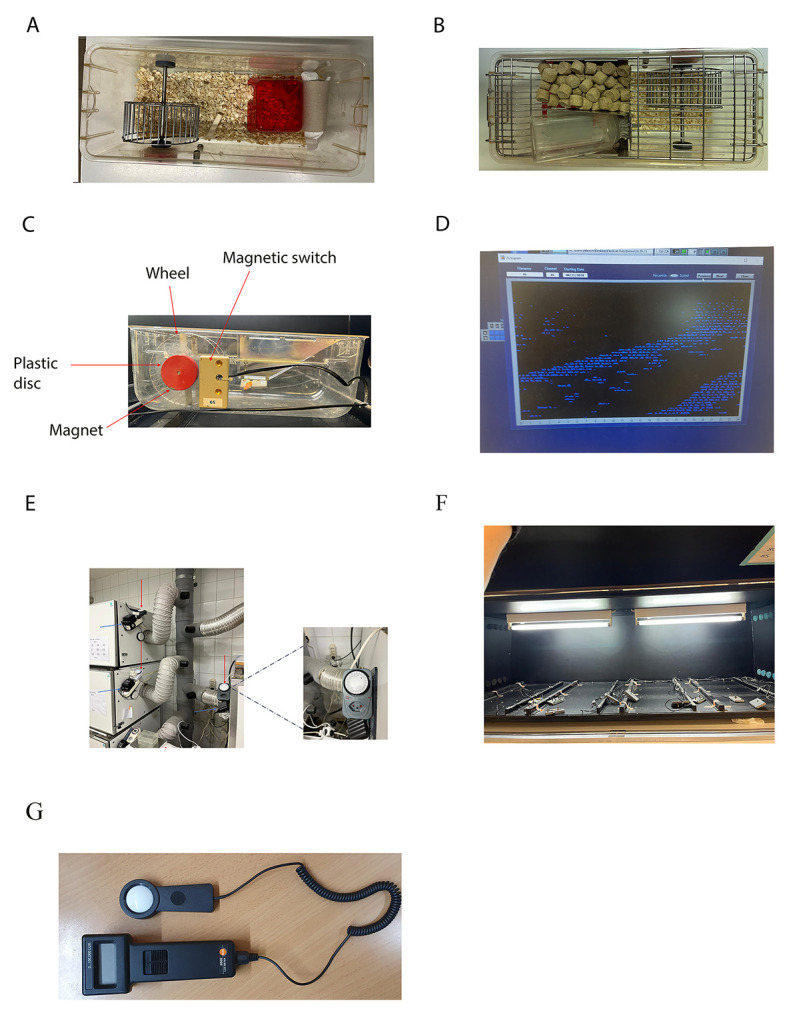
Essential tools for the experimental procedure. **A)** Representative cage with nesting and bedding materials. **B)** A photo of the cage showing the food and water configuration. **C)** Example of a wheel-running cage connected via a magnetic switch to the system recording wheel revolutions. On each rotation of the running wheel, the magnet, embedded in the plastic disc, activates the magnetic switch, opening and closing. The magnetic switch cable sends the info to the computer, which elaborates the data displaying the number of wheel-running revolutions in the actogram. **D)** Representative actogram displayed on the computer when the info obtained from the magnetic switch is elaborated by ClockLab. **E)** The external timer allows the user to set the appropriate light/dark cycle, avoiding multiple cabinet openings, which might eventually drop the internal temperature. The red arrows point to the timers. The blue arrows point to the power generator. **F)** Example of a cabinet interior and illuminating system. **G)** Example of a luxmeter that can be used for measuring light intensity.

1. Plastic cages with steel running wheels must be prepared with bedding and nesting material (General note 1) (Figure 2A).

2. Weigh mice between 3 and 5 months old and check to evaluate their health (General note 2). Weight and health conditions should be noted on an appropriate scoresheet approved by the veterinary office (Document S1).

3. House mice individually in wheel-running cages with access to food and water ad libitum (Figure 2B).

4. Wheel-running cages are provided with a small magnet nestled in a plastic disc that rotates when the wheel moves (Figure 2C). The signal is transmitted to the computer through a connector plugged close to the plastic disc, and the rotations are visualized by the clock lab software (Figure 2D). Plug the connector and test the magnet before starting the experiment (General note 3). Each cabinet can contain a maximum of 12 boxes. Cabinets are soundproof and ventilated (General note 4).

5. Set the timer to have light/dark (LD) cycles of 12:12 h. The timer is mounted outside the cabinet (Figure 2E). Lock the cabinets (Figure 2E) and start the recording. The illumination is ensured by two light bulbs (1,000 lux, Figure 2F) mounted on the ceiling of the cabinet (General notes 5 and 6). The light intensity is confirmed by a luxmeter (Figure 2G).

6. During the first day of the current experiment, conditions (L:D, D:D), number of recorded days, mouse genotype, age, and sex need to be annotated before the experiment into an appropriate sheet (Document S2).

7. The experimenter should be particularly careful in monitoring mice activity during the first days (General note 7).

**B. Wheel running activity recording and validation of the free-running period**

1. Mice should be entrained to the L:D 12:12 cycles for at least 10 days to adapt to the isolation cabinet and the diurnal cycle. At this stage, the entire activity will be confined to nighttime since mice are nocturnal animals, and the actogram will show two separate phases: a resting (*rho*) and an active phase (*alpha*) (Figure 1B).

2. After at least 10 days, mice can be released in constant darkness, and a free period will appear on the actogram like a slope (Figure 1C). If the experimenter wants to measure only the free period, they can turn off the illuminating system anytime, after ZT12. Turning the light off after ZT12 would allow mice to enter the free-running period the day after. The illuminating system should be turned off at ZT10 to validate the Aschoff-type II protocol.

3. The free-running period should last at least 10 days. Once a stable free-running rhythm is established, the experimenter can determine the internal period length and the overall activity as revolutions/day. The free-running period of wild-type mice should be less than 24 h. Since the computer clock is set to 24 h, the free running will appear on the actogram like a slope (Figure 2D).

4. Mice are re-entrained to the light/dark cycle for at least 10 days (i.e., Figure 1D) before being ready for the phase shift of the circadian clock protocol.

**C. Phase shift of the circadian clock**

**Figure 3.**
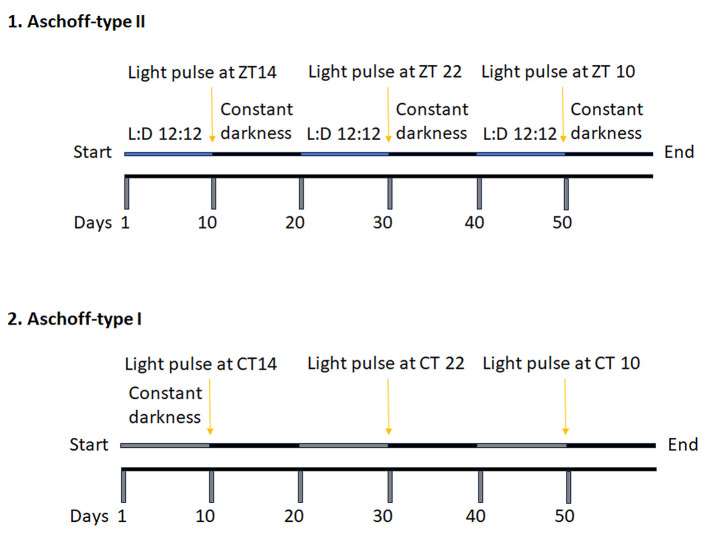
Schematic representation of the Aschoff-type II and Aschoff-type I protocol for measuring the phase shift of the circadian clock. After ten days under diurnal conditions (blue line; L:D 12:12), mice receive light exposure (Aschoff-type II) at ZT14 (2 h after lights off) and ZT22 (2 h before lights on). The control at ZT10 can be achieved simply by switching off the light at that specific time point. Following the light pulse, mice are kept in constant darkness for ten days (free running, black line). After ten days under circadian conditions (grey line), the appropriate CT14, CT22, and CT10 are determined, and light is applied (Aschoff-type I). Mice are then monitored for another ten days in constant darkness (black line).

The phase shift of the circadian clock can be measured with two different protocols: Aschoff-type II and Aschoff-type I. Applying the type II protocol, we give light pulse at ZT14 and ZT22 followed by releasing mice in constant darkness and measure the angle between the free-running and diurnal periods. Applying the type I protocol, mice are already released in constant darkness. Therefore, we measure the endogenous circadian hour and give the light pulse at CTs 14, 22, and 10. A schematic example of the protocol is shown in Figure 3. For more details about how to calculate the proper CT, please see the data analysis section.

Why use two different protocols? The Aschoff-type I can be applied to animals with a stable free-running rhythm when kept in constant darkness. We can apply only the Aschoff-type II protocol when animals show arrhythmic free running (i.e., *Per1*ko/*Per2*ko mice). Due to the arrhythmicity, it is impossible to calculate the CT based on the endogenous circadian hour. Additionally, for fast screening of mutant animals that might display aberrant phase shift responses to light pulses, the Aschoff-type II protocol is easier to apply because we can give the light pulse to all animals simultaneously. However, if mutant mice show a regular free-running period, the Aschoff-type I protocol needed to be applied as well. The advantage of the type I protocol is that the phase shifts can be easily determined as the difference between two almost parallel lines. However, for every individual animal, the time of light application has to be calculated based on its period (see below).

**Aschoff-type II**

1. Mice should be entrained into the L:D 12:12 cycles for at least 10 days to adapt to the isolation cabinet and the diurnal cycle. After 10 days, 15’ of a light pulse can be applied at ZT14 (phase delay), and mice can be released for another 10 days in constant darkness to show a stable free-running period.

2. Mice are re-entrained into the light/dark cycle for at least 10 days before being ready for the phase shift of the circadian clock protocol.

3. After the re-entrainment, 15’ of a light pulse can be applied at ZT22 (phase advance), and mice can be released for another 10 days in constant darkness to show a stable free-running period.

4. Mice are re-entrained into the light/dark cycle for at least 10 days before being ready for the phase shift of the circadian clock protocol.

5. After the re-entrainment, 15’ mice can be released for another 10 days in constant darkness directly at ZT 10 (no phase delay).

**Aschoff-type I**

1. The circadian hour will be calculated based on the circadian free-running period (see the next section) obtained from the previous protocol (Aschoff-type II, step 5). Then, the specific time point will be calculated based on the CT.

2. 15’ of a light pulse can be applied at CT14 (phase delay), and mice can be kept for another 10 days in constant darkness.

3. An additional 10 days of constant darkness ensures that the effect of the previous light pulse is eliminated.

4. 15’ of a light pulse can be applied at CT22 (phase advance), and mice can be kept for another 10 days in constant darkness.

5. An additional 10 days of constant darkness ensures that the effect of the previous light pulse is eliminated.

6. 15’ of a light pulse can be applied at CT10 (no phase delay), and mice can be kept for another 10 days in constant darkness.

7. Mice can be re-entered in a light/dark cycle if they are needed for other experiments.

## Data analysis

**A. How to open an actogram file in Clock Lab**

**Figure 4.**
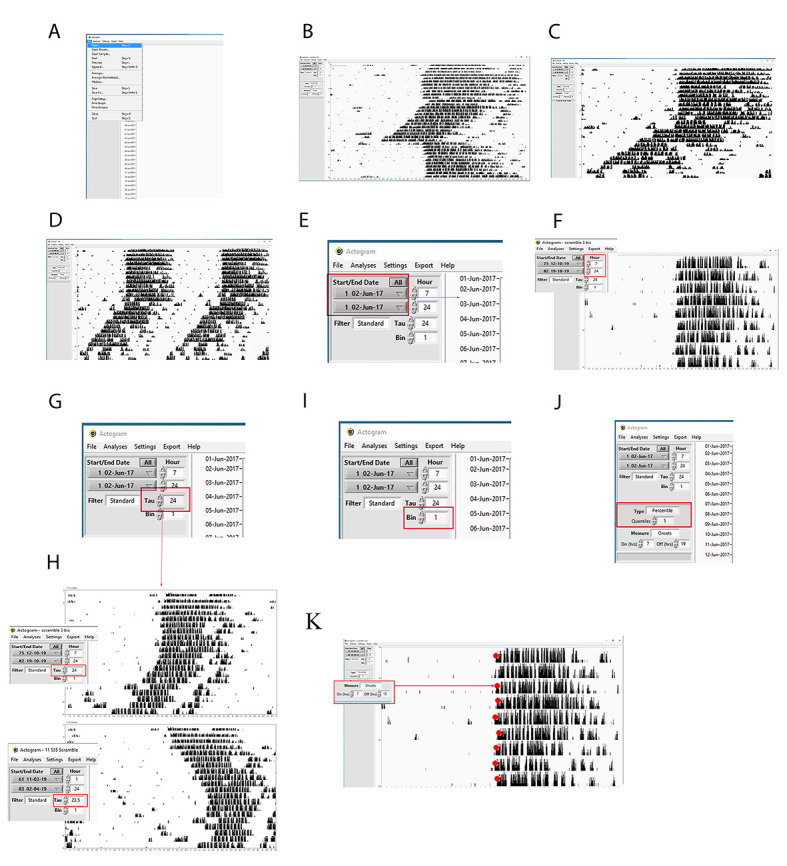
Step-by-step guide for measuring chronobiological parameters using ClockLab. **A, B)** Representative screenshots showing how to load an actogram on ClockLab. **C)** Difference between single and **D)** double-plotted actogram. **E)** Representative screenshot showing how to select the days for analyzing data. **F)** Representative screenshot showing how to center the actogram to have half of it showing the light phase and half of it showing the dark phase. In this case, the light was on at 07:00 and off at 19:00; therefore, setting 07:00 in the hour box would let the actogram start when the light is on, and the onset activity would appear in the middle of it. **G)** Representative screenshot showing how to select the tau. Tau:24 means that the mice profile defined by their wheel-running activity is distributed over 24 h. **H)** Example showing how manipulating the tau can change the shape of the actogram. If the tau is set to 24 h, the actogram looks straight in diurnal conditions and as a slope with anticipating onset in circadian conditions. If we set the tau at the circadian period in constant darkness, then the actogram in diurnal conditions will look like a slope delaying the onset every day, while the actogram in constant darkness will look straight. **I)** Representative screenshot showing how to select the window of time displaying the number of wheel-running revolutions. Bin:1 means how many revolutions per minute. **J)** Representative screenshot showing how to modify the appearance of the actogram by selecting the more appropriate “type.” **K)** Representative screenshot showing how to select the onset and offset time. The red dots (their size was increased to make them visible) displayed on the actogram onset of the mouse activity profile for each day are adjusted accordingly.

1. Open Clock Lab.

2. Go to *Open*, search for the actogram file that can be read by ClockLab, and click on it (Figure 4A).

3. The actogram appears in the Clock Lab (Figure 4B).

4. The user can choose between a single (Figure 4C) and a double (Figure 4D) plot actogram. To double-plot the actograms, the user can go to the tool settings and select double-plotting.

**B. Analysis controls**

Start/end dates: The menu contains a list of the dates (month-day-year) for which data were collected, together with the number of each day that appears on the actogram on the Y-axis (Figure 4E). These menus can be used to set the range of data from which all calculations and graphs are made.

Start/end hour: They determine the time frame window (hours) within which the actograms are analyzed. This control is particularly important for the actogram display. The user can set the hour (i.e., hour 1: 7:00; hour 2: 24:00) to match the onset and offset (i.e., 7:00 light on, 19:00 light off) to have the *rho* and *alpha* phases properly distributed (Figure 4F).

Tau: It influences the appearance of the actogram and the activity profile. The default Tau is exactly 24 h (Figure 4G). If the Tau is set with the time corresponding to the mouse free-running period, the actogram will not appear like a slope anymore, but it will look straight and L:D will look like an advanced slope (Figure 4H).

Bins: The user can set the bin width, namely the window of time taken into account for measuring the wheel-running revolutions (Figure 4I).

Type: Four types of actograms can be displayed (percentile distribution, even distribution, threshold, scaled). The choice of the type will influence the appearance and size of the black bars associated with the count of wheel-running activity (Figure 4J). For further details, please visit the appropriate website (https://actimetrics.com/products/clocklab/).

Onset/offset: The red dots show the results of the automated wheel-running activity onset. They are necessary indicators for drawing the regression line used for measuring the internal period length. Given the onset and offset time, the software can calculate at a rate of 95% correctly for most data. If the red dot is misplaced, it can be corrected simply by shift-clicking on the appropriate point within the line for that day (Figure 4K).

**C. Features**

1. Period length

**Figure 5.**
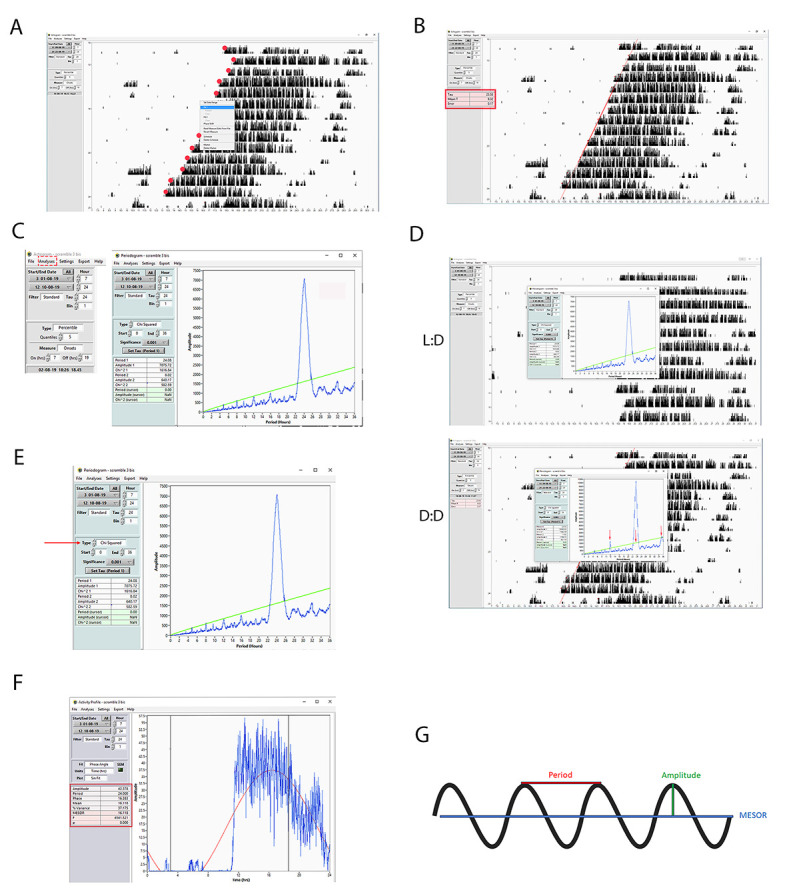
Step-by-step guide for measuring the circadian period length using ClockLab. **A, B)** Screenshots showing how to measure the period length based on the activity onset calculated on the assigned red dots (their size was increased to make them visible) of eight following days. **C)** Screenshot showing a representative periodogram selected from the analysis tool. **D)** Example of two different periodograms. The upper one displays the period length of a mouse kept under diurnal conditions (12:12 h light/dark), and the lower one displays the period length of the same mouse kept under circadian conditions (constant darkness). The red arrows indicate ultradian phenotypes that appear with a periodicity of 12 h (12, 24, 36 h). Note that at 24 h, the phenotype is masked by the dominant circadian period. The green line indicates *Chi-squared*. **E)** Screenshot showing how to select the appropriate statistical test for the periodogram. In the figure, the *Chi-squared* periodogram is shown. **F)** Screenshot showing a representative activity profile graph of a diurnal mouse. An activity profile is an option contained in the analysis tool. In the red box, all parameters analyzed are displayed. **G)** A schematic representation that shows the graphical meaning of some relevant parameters like the period, MESOR, and amplitude. For the explanation, read the appropriate section in the text.

Mice must be kept for at least 10 days in constant darkness to quantify the free-running period length (τ). Then, the red dots displaying the activity onset need to be properly assigned at the beginning of the daily wheel-running activity in constant darkness. Subsequently, a right-click on the mouse will show a drop-down menu (Figure 5A). The user should click **fit1**. The tool generates a least-squares fit to a group of the points (each point is one day) displayed in the actogram window. Finally, it draws a line of the selected following days of activity, excluding the first two (transient cycles). The endogenous period length (τ) will be determined from the regression line drawn through the activity onsets [17] (Figure 5B). Along with the fitted line and tau, we can analyze (Figure 5B, red box):

**• Error:** The standard deviation of the horizontal distance between the onset activity points and the regression line.

**• Mean:** The mean time in hours calculated for the regression line, considering the number of days taken into account for calculating the tau.

The calculated period length can be used to compare different genotypes or pharmacological conditions.

2. Periodogram

A periodogram, namely the periodogram of Enright [18], consists of an analysis employed for short-term rhythms. It represents one of the options offered by ClockLab for measuring accurately the internal period length. The periodogram is calculated within a period set by the tau (i.e., 24 h) displayed on the x-axis of the graph. The data are cut into segmental periods (i.e., data analyzed within a window of two consecutive hours) within 24 h. The same segments, also called modules, from several days of analysis (at least seven consecutive days) are averaged together to give an activity profile, and the standard deviation of the profile is calculated. Following the Enright model, circadian data are plotted to provide a quotient of variance across the different modulo segments. Each modulo corresponds to a specific circadian period. The modulo with the largest value determines the dominant period length. The user should select from the “start-end” boxes the range of days that have to be analyzed. Then, on the main window, select *analysis* and finally *periodogram* (Figure 5C). The blue trace will show peaks of activity calculated by averaging several days within each segment of two hours composing the entire tau (24 h). The highest peak will determine the period length. Of note, we reported two cases—one where the periodogram measured the period in constant darkness (23.55 h) and the other where the period was measured in L:D conditions (24.05) (Figure 5D). The mouse analyzed was the same. Together, these results indicate that the periodogram is an efficient tool for precisely measuring the internal period. The importance of this model is given by the fact that it can also display ultradian rhythms [19] compared to the least-squares regression fit (Figure 5B), recurrent periods, or cycles repeated throughout a 24-h day. Figure 5D indicates with red arrows a recurring ultradian rhythm with a period of circa 12 h (masked by the circadian rhythm at 24 h). There are three different statistical analyses based on different formulas or algorithms used for implementing the calculation of the internal period length within a periodogram. These can be selected by clicking on *Type* (Figure 5E, red arrow):

• *Chi-squared (χ^2^) periodogram*: It is calculated after the method of Sokolove and Bushell [20]. *χ^2 ^* is defined as a ratio of the variance observed at a specific period segment compared to the total average variance in the data set. *χ^2^* values increase with the amplitude of the rhythm. If the amplitude is generally low, the *χ^2^* becomes a less sensitive tool.

• *Fourier-periodogram (F)*: It is calculated after the method of Dörrscheidt and Beck [21]. F analysis implies that any modulo can be approximated by a series of sine and cosine waves of differing period, amplitude, and phase. Therefore, the modulo is transformed into a series of simple waveforms, with coefficients determined based on the goodness of fit to the data.

• *Lomb-Scargle periodogram*: It is calculated after the method of Lomb [22]. It is an adaptation of the F-periodogram for datasets with missing values or irregular data [23].

The user can select the statistical analysis (chi-square, F, Lomb-scale) on the box type. In our example, the selected chi-square will appear as a green line (Figure 5D), and the period is measured considering the spectrum that is crossing the *Chi-squared* line.

3. Activity profile

The activity profile displays the distribution of the activity onsets (wheel-running revolutions counts) over 24 h, averaging the range of days taken into account. The user can obtain an activity profile of the selected range of days, by clicking on *Analysis* and then *Activity profile*. The software will calculate the distribution of the mouse's general activity divided over 24 h. As shown in Figure 5F, in diurnal conditions, the mouse is active starting from ZT12 to ZT0 (*alpha* phase) and resting from ZT0 to ZT 12 (*rho* phase). Interestingly, as mentioned before, during the resting phase, mice can show scattered activity, which is also displayed by the activity profile. On the other hand, between ZT18 and ZT22, mice show a “siesta” moment, where their activity drops before starting again until the end of the dark phase. The shading around the activity profile trace indicates the standard deviation for each point.

Users can find in the activity profile menu the following items (Figure 5F, red box):

**• Fit:** Select the phase angle to have the activity profile phased to the 24 h (solar day).

**• Units:** The user can set the x-axis in degrees (t/Tau*360), hours, circadian hours, %, or Tau.

**• Sin fit:** The red line is a sine fit to the waveform. Parameters describing the fit are shown in the table to the left, including amplitude, phase, and mean.

**• Amplitude:** Delta between MESOR (mean of the circadian rhythm) and the peak of the sinusoidal-shaped circadian rhythm (Figure 5G).

**• Period (24 h):** The period is used for measuring the phase angle (it is usually 24 h). The period is the distance between two peaks of the sinusoidal-shaped circadian rhythm (Figure 5G).

**• MESOR:** Midline statistic of rhythm; it is a rhythm-adjusted mean to the individual rhythm (Figure 5G).

**• F statistic:** It represents a measure of the robustness of circadian activity rhythm. Increasing values indicate a stronger rhythm, whereas decreasing values indicate a weaker rhythm.

**• Estimate of p-value:** For such a robust rhythm as shown here, it is arbitrarily close to zero.

**• Mean:** Places a horizontal line in the activity profile at the mean or median of the graph. This control also determines whether a mean or median line appears in a batch printing of activity profiles.

**• Export:** Export is part of the activity profile analysis tool. It displays counts for each day in the MATLAB command window and saves the counts to a spreadsheet-compatible file like Excel. The file also contains the raw data (time and counts/day) of the activity profile plot.

4. Phase shift

a. Aschoff-type II

**Figure 6.**
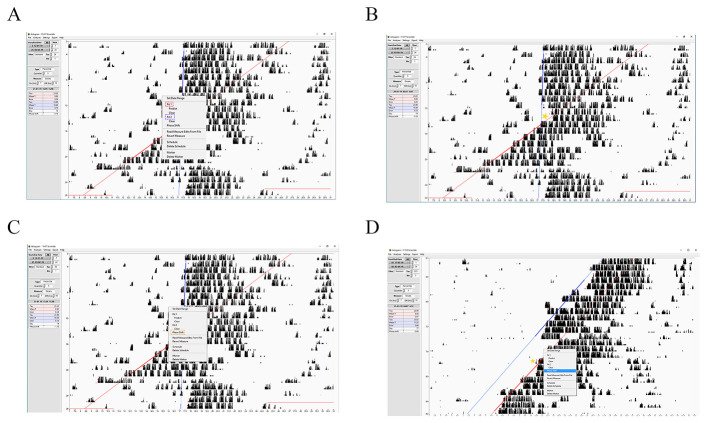
Step-by-step guide for measuring the phase shift of the circadian clock using ClockLab. A–C) Representative screenshots showing how to calculate the phase shift of the circadian clock when the Aschoff-type II protocol is applied. **D)** Representative screenshots showing how to calculate the phase shift of the circadian clock when the Aschoff-type I protocol is applied. The yellow stars indicate where to point the cursor before clicking *phase shift*.

The actogram should display at least 10 consecutive days in diurnal and 10 consecutive days in circadian conditions. When the onset activity time is set for mice in both L:D and constant darkness conditions, the least-squares regression fits the onsets that have been calculated for the diurnal and circadian rhythms. Usually, with **fit1**, we draw a regression line for the following days in constant darkness, excluding the first two days to avoid the effect caused by the transition cycles. The **fit2** regression line is dragged to the seven following days of the previous diurnal cycle (Figure 6A). When the user measures the phase shift, they need to be sure to point the mouse cursor at the part of the angle corresponding to the second day after the light pulse (yellow star, Figure 6B) to have a better-optimized phase shift. Then the user can move the cursor to the angle formed by the two regression lines and click on phase shift (Figure 6C).

b. Calculation CT

To perform the Aschoff-type I protocol, it is necessary to calculate the subjective phases (CT, or circadian time) of an animal’s rhythm based on the individual organism’s free-running rhythm. To that extent, the first thing to do is to calculate the circadian hour by dividing the internal period length τ (see section C1 of Data analysis) by 24 the day before the phase shift experiment (Day A):

1 circadian hour = τ/24

For instance, if the free-running period is 23.7 h, calculated in Day A, the circadian hour will be 0.987 (23.7/24). It has to be taken into account that for each day spent in constant darkness, CT12 diverges further from ZT12. On the first day in constant darkness, we can assume they are almost the same, but each day, the divergence between the internal and external tau increases (i.e., 24 - 23.7 = 0.3 h of divergence for each day). Thus, to be precise about calculating the proper CT10, CT14, and CT22, we can proceed as follows.

• The researcher has to calculate the CT12 of the next day (Day B) by applying the following formula:

CT12 Day B = CT12 Day A + τ - 24 h

For instance: CT12 Day B = (0.987*12) + 23.7 - 24 h → CT12 = 11.544 h

• To define a circadian time from CT0 to CT12, one can apply the following formula:

CTX_0-12_ = CT12_Day B_ - (X*1 circadian hour) → [X = CT12 - CTx]

For instance: CT10 = 11.544 - [(12 - 10)*0.987] → CT10 = 9.57 h

• To define a circadian time from CT12 to CT24, one can apply the following formula:

CTX_12-24_ = CT12_Day B _+ (X*1 circadian hour) → [X = CTx - CT12]

For instance: CT14 = 11.544 + [(14 - 12)*0.987] → CT14 = 13.518 h

**Aschoff-type I**

The actogram should display at least 10 consecutive days in constant darkness before and after the light pulse. When the onset activity time is set for mice before and after the light pulse, the least-squares regression fits the onsets that have been calculated (we proceed as we did for the Aschoff-type II protocol). We double-click on the second day after the light pulse, and we calculate the phase shift (yellow star, Figure 6D). It is important to note that it is easier to visualize the phase shift of the circadian clock applying the Aschoff-type I protocol since when the phase shift happens, **fit1** and **fit2** run parallel (Figure 6D).

**Statistical analysis**

Statistical analysis can be performed using GraphPad Prism6 software. Depending on the data type, either an unpaired t-test or one- or two-way ANOVA with Bonferroni or Tukey’s post-hoc test can be performed. Values considered significantly different are highlighted [p < 0.05 (*), p < 0.01 (**), or p < 0.001 (***)].

Data compared via one-way ANOVA with post hoc Bonferroni’s corrections requires repeated-measures multiple comparisons among columns. Data (normal) distribution must be assessed, and accordingly, parametric or non-parametric tests must be applied. All data must display the mean ± standard error of the mean (SEM) and individual values. The suggested number of mice should be at least six per experimental group.

## Validation of protocol

This protocol or parts of it have been used and validated in the following research articles (among others):

• Brenna et al. [24]. Cyclin-dependent kinase 5 (CDK5) regulates the circadian clock. *eLife*. (Figure 2)

• Brenna et al. [16]. Cyclin-dependent kinase 5 (Cdk5) activity is modulated by light and gates rapid phase shifts of the circadian clock. *eLife.* (Figure 1)

• Chavan et al. [25]. Liver-derived ketone bodies are necessary for food anticipation. *Nat Commun*. (Suppl. Figure 2)

• Schmutz et al. [26]. A specific role for the REV-ERBα-controlled L-Type Voltage-Gated Calcium Channel CaV1.2 in resetting the circadian clock late at night. *J Biol Rhythms*. (Figures 1 and 2)

**Advantages and disadvantages**

To date, the wheel-running activity is still the best method for extrapolating important information about diurnal/circadian behaviors such as free-running period, phase angle of entrainment to the LD cycle, period amplitude, and daytime running activity. Although numerous methods exist to measure the phase shift of the circadian clock (i.e., monitoring general activity using photo beams or the core body temperature by implanting sensors [27]), the wheel-running activity is still the less invasive approach. Compared to the general activity measurement via photo beams or core temperature, wheel-running measures only voluntary activity and not other activities such as grooming. Due to this, the wheel-running shows clearer onsets and offsets of activity, making the interpretation of an actogram easier. However, there are several points to take into consideration. The lighting conditions should be adjusted via a timer without opening the box. The isolation cabinets are well-ventilated to avoid overheating. However, light is a stronger and more immediate Zeitgeber than temperature. Therefore, the observed temperature variations under LD conditions can be neglected. Under constant darkness conditions, the temperature excursion during the day should be minimal since environmental temperature cycles can sustain peripheral circadian clocks [28]. Mice are kept in single caging for a certain amount of time. Although individual cages are close to others, and mice can still smell each other, long exposition to solitude might eventually affect their mood (i.e., stereotypical behaviors). ClockLab provides a wide range of options for measuring all the circadian parameters described above. However, any option comes with strengths and weaknesses. Please read the review [29], which gives more insights about the topic.

## General notes and troubleshooting

**General notes**

1. The experimenter should not use excessive bedding to avoid wheel blockage while the mouse is on it.

2. It is important that age, gender, and weight match as much as possible to avoid variability that might affect the mouse’s wheel-running activity [30–32].

3. Once the connector is plugged into the cage, manually rotate the wheel and check on the computer if the ClockLab software is counting the revolutions.

4. Please turn off the room's light and check whether the cabinet is properly isolated (no light from the inside should be visible).

5. The light intensity is an essential factor. Therefore, confirm the proper light intensity using a luxmeter. Regular gray/black mice can perceive light up to 1,000 lux. Albino mice should be treated more carefully since they are more sensitive to light. The color temperature of the bulb is an important factor, measuring the visual “whiteness” of the light, and its unit is degrees Kelvin (K). Blue light, which is cold, on the other hand, displays a high color temperature and ranges. The necessary signal for entraining mice to the light/dark cycle is contained in the white light with natural daylight at temperatures between 6,000 and 7,000 K. The bulb described above has a luminous flux of 1,000 lumens and a color temperature of 6,500 K.

6. The time set on the timer should correspond to the actual zone time corresponding to the experimenter's location.

7. During the first three days of the experiment, the experimenter should monitor the actogram produced by the ClockLab software to ensure that all the connectors are recording and the proper light and dark conditions are working fine.

**Troubleshooting**

Problem 1: Light dispersion from the cabinet.

Possible cause: Door seals damaged.

Solution: Replace door seals.

Problem 2: The computer is not detecting wheel-running revolutions.

Possible cause: The magnet or magnetic switch is damaged.

Solution: Replace the magnet or magnetic switch.

Problem 3: The computer displays few or no wheel-running activity for a specific mouse on the actogram.

Possible cause: The wheel is stuck with bedding, or the mouse is sick.

Solution: Open the cage during the light phase and check whether the problem is the wheel or the mouse.

Problem 4: The computer displays activity bouts anticipating or delaying the external onset/offset (light on/light off).

Possible cause: The timer is not working properly

Solution: Check at the time of day when the light should be turned on and off to confirm that the timer is working. If it is not working, replace it.

Problem 5: The phase shift of the circadian clock (Aschoff-type I) did not work as expected in wild-type mice.

Possible cause: The CT calculation was wrong.

Solution: Check all the parameters again and perform new calculations.

## Supplementary information

The following supporting information can be downloaded here:

1. Document S1. Score sheet

2. Document S2. Wheel running datasheet
